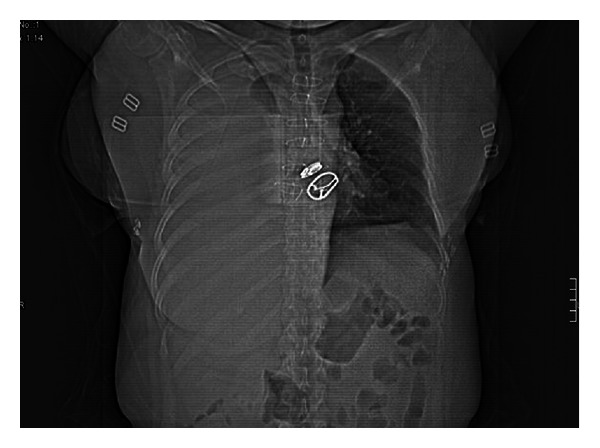# Nontraumatic Massive Spontaneous Hemothorax with Concomitant Warfarin Use

**DOI:** 10.1155/2013/546024

**Published:** 2013-05-12

**Authors:** Nurettin Özgür Doğan, Gül Pamukçu Günaydın, Mustafa Tekin, Yunsur Çevik

**Affiliations:** ^1^Department of Emergency Medicine, Faculty of Medicine, Kocaeli University, Kocaeli, Turkey; ^2^Department of Emergency Medicine, Ankara Atatürk Training and Research Hospital, Ankara, Turkey; ^3^Department of Emergency Medicine, Keçiören Training and Research Hospital, Ankara, Turkey

## Abstract

Hemorrhagic complications due to warfarin use are frequently seen in emergency departments. However, nontraumatic massive hemothorax is an unexpected complication. We report a 59-year-old woman with warfarin overdose, who had massive hemothorax in right lung without any history of trauma. Her main complaint was significant dyspnea, which has gradually increased in three days. On her physical examination, she was tachypneic and had decreased lung sounds on her right hemithorax. She took warfarin regularly for aortic and mitral valve replacement for 18 years. Her INR level was 12.9 (0.8–1.2). Computed tomography of thorax revealed massive hemothorax with mediastinal shift. Fresh frozen plasma infusion was started immediately. Tube thoracostomy was performed for reexpansion of right lung and 2000 cc blood was drained in 5 minutes. Although hemorrhagic complications can be expected in warfarin therapy, thoracic hemorrhage related to warfarin therapy is relatively rare (3% of all hemorrhagic complications due to warfarin therapy). To our knowledge, massive hemothorax due to warfarin use is an extremely rare condition.

## 1. Introduction

Warfarin is a commonly used oral anticoagulant; warfarin therapy is associated with a number of adverse drug reactions including bleeding. Important risk factors for major hemorrhage due to warfarin therapy include history of gastrointestinal bleeding, concurrent use of antiplatelet or nonsteroidal anti-inflammatory drugs, genetic differences in warfarin metabolism, INR variability, comorbid illnesses, and duration of oral anticoagulant therapy [1]. Thoracic hemorrhage accounts for approximately 3% of all hemorrhagic complications associated with warfarin therapy and is usually related to trauma [2].

Hemothorax due to warfarin therapy is a relatively rare complication, and trauma is a major risk factor. We reported a 59-year-old woman with warfarin overdose, who developed massive hemothorax in her right lung without having any history of trauma. To our knowledge, massive hemothorax due to warfarin use is an extremely rare condition.

## 2. Case Presentation

A 59-year-old woman presented to emergency department with dyspnea, which has gradually increased in three days. She did not complain of having any fever, cough, or sputum, but she had right-sided pleuritic chest pain. She has been taking warfarin for aortic and mitral valve replacement for 18 years and her INR levels were checked regularly. Additionally, she used 100 mg aspirin and an angiotensin receptor blocker daily. In her medical history, she did not have any systemic illnesses other than hypertension and she did not remember any kind of trauma during last month including any minor trauma. 

Her body temperature was 36.8°C, blood pressure was 100/70 mmHg, oxygen saturation was 88%, and heart rate was 96 beats per minute; it was irregular with atrial fibrillation. On physical examination, her lung sounds were globally decreased at right hemithorax and she was tachypneic. Physical examination of other systems was unremarkable, except for metallic valve sounds which were heard on auscultation from aortic and mitral valves.

**Figure 1:**
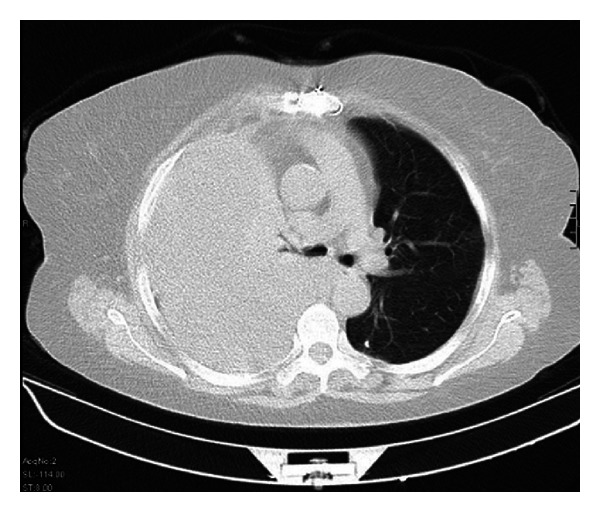


In her laboratory findings, her complete blood count, liver and renal function tests, and electrolyte levels were normal; serum *β*-human chorionic gonadotrophin was negative. Her INR level was 12.9 (0.8–1.2). The chest X-ray of the patient revealed a large pleural effusion in right hemithorax, which was not evident in her previous X-rays. The computed tomography of her thorax revealed massive right-sided hemothorax with mediastinal shift to the left side (Figures 1 and 2). Vascular structures were normal. No other mass lesions or infectious lesions were detected. Her medical history was asked again with particular attention to any trauma, and she denied having any trauma including minor trauma in the previous month.

A tube thoracostomy was performed for reexpansion of right lung and the patient was given 18 mg/kg fresh frozen plasma infusion simultaneously. 2000 cc blood was drained in 5 minutes by insertion of chest tube. Bloody drainage was confirmed via hematologic assay. She was supported with 1000 mL intravenous saline and no hypotension was observed except for a brief time interval during tube thoracostomy.

The patient was admitted to intensive care unit and decrease in bloody drainage was observed in following days. Samples of the pleural fluid were sent for cultures and Cytologic examination. The cultures of the pleural fluid and sputum revealed no infectious disease including tuberculosis. The Cytological examination was negative for malignant causes. Chest tube was removed after 5 days; there were no complications. She was discharged home, after appropriate dose adjustments for warfarin have been made. In her followup after one month, she had therapeutic INR levels and no effusion was seen in her chest X-ray.

## 3. Discussion

Warfarin is an important drug, which was used for different clinical entities including atrial fibrillation, heart valve replacement, venous thrombosis, and pulmonary embolism. It is one of the risky drugs, which was constituted as a significant part of emergency department presentations due to drug overdose in United States [3]. 

Hemothorax is very rare in the setting of anticoagulation, and usually occurs within the first week of therapy [4]. Our patient took warfarin 18 years ago and since then she did not experience any important hemorrhagic event. Her INR levels were in the therapeutic range since she was anticoagulated. In the literature, only one case with spontaneous hemothorax related to artificial heart valve was reported; however, accumulated blood in that case was not massive [5]. 

A similar case reported by Çiledağ et al. revealed spontaneous hemothorax due to warfarin therapy in the setting of atrial fibrillation, but their patient was managed conservatively owing to few blood depositions in the pleural space [6]. Pulmonary diseases including pleural pathologies were considered significant risk factors for developing nontraumatic hemothorax in the setting of anticoagulation. However, our case had no previous pulmonary disease including pleural or pulmonary malignancies and pulmonary embolism; also no aortic dissection or other hematologic conditions were diagnosed.

Low-molecular-weight heparins could also be responsible for spontaneous hemothorax during therapy. In a case described by Mrug et al., a 58-year-old woman had spontaneous bilateral hemothorax after four days of anticoagulation therapy with enoxaparin for suspected pulmonary thromboembolism. The case was managed with red blood cell and plasma transfusions, bronchodilators, and repeated thoracenteses. Standard tube thoracostomy procedure was not performed [7]. This is a unique case in the literature of spontaneous hemothorax with concomitant enoxaparin use.

Hemothorax is a major indication for tube thoracostomy, particularly in cases with mediastinal shift. However, accelerated drainage may cause significant hypotension and supratherapeutic INR levels should be evaluated carefully. In our case, we decided to perform tube thoracostomy simultaneously with fresh frozen plasma infusion due to significant mediastinal shift. Although warfarin is considered as a cornerstone therapy in numerous conditions, hemorrhagic complications should be cautiously handled.

## Figures and Tables

**Figure 2:**